# Effect of Peanut Shell and Rice Husk Bedding for Dairy Cows: An Analysis of Material Properties and Colostrum Microbiota

**DOI:** 10.3390/ani12050603

**Published:** 2022-02-28

**Authors:** Pengtao Li, Tong Fu, Amin Cai, Kris Descovich, Hongxia Lian, Tengyun Gao, Clive J. C. Phillips

**Affiliations:** 1 College of Animal Science and Technology, Henan Agricultural University, Zhengzhou 450046, China; pengtao_li@126.com (P.L.); futong2004@126.com (T.F.); amin_cai@163.com (A.C.); lhx263@sina.com (H.L.); 2 Center for Animal Welfare and Ethics, School of Veterinary Science, The University of Queensland, Gatton, QLD 4343, Australia; k.descovich1@uq.edu.au; 3 Sustainable Policy (CUSP) Institute, Curtin University, Bentley, WA 6102, Australia

**Keywords:** dairy cow, properties, colostrum microbiota, peanut shell, rice husk

## Abstract

**Simple Summary:**

The provision of appropriate bedding is important for the welfare of dairy cows. Before bedding can be selected, it is critical to understand the properties of the bedding, including its impact on milk microbiota. The objective of this article was to evaluate the influence of three materials for use as bedding on physicochemical properties, bacterial counts and colostrum microbiota of cows. Our results demonstrate that peanut shells appear to be a suitable bedding material for cows. These experiments provide empirical support for the use of peanut shells and rice husks as bedding material for dairy cows and illustrates the effects of bedding types on the colostrum microbiota of dairy cows.

**Abstract:**

The aim of this study was to evaluate peanut shells and rice husks as bedding for dairy cows. We analyzed material properties including dry matter, water holding capacity, pH level and bacterial counts. Bedding treatments were compared with a one-way ANOVA using twelve cows split into three groups. Colostrum microbiota was analyzed by sequencing of the V3–V4 region of the 16S rRNA gene. Dry matter content was higher in rice husks compared with peanut shells. No treatment effects were found for water holding capacity and pH level. *Streptococcus agalactia* counts in peanut shell bedding were lower than in rice husk bedding, and *Pseudomonas aeruginosa* counts were not different between beddings. A significant enrichment for *Enhydrobacter* and *Pantoea* were detected in the colostrum of cows that used peanut shells compared with other beddings. Colostrum of cows housed on a peanut–rice combination had a greater relative abundance of *Pseudomonas* and *Corynebacterium* than those housed on peanut shells or rice husks. Higher numbers of *Bacteroides*, *Akkermansia*, *Alistipes*, *Ruminococcaceae_UCG-014*, *Coriobacteriaceae_UCG-002* and *Intestinimona* were found in the colostrum of cows housed on rice husk bedding over other bedding types. These results suggest that bedding types were associated with the growth and diversity of colostrum bacterial loads. In addition, dry matter in peanut shells was lower than found in rice husks, but there was also a lower risk of mastitis for peanut shell bedding than other beddings.

## 1. Introduction

Bedding plays an important role in the welfare of housed dairy cows [1], as they usually lie on it for 8–16 h/d to rest [2]. Good bedding improves cows’ cleanliness, behavior, and udder health, and reduces hoof injuries [3,4,5]. Cows lie down for longer when the bedding is dry, soft and clean [2,3,6]. Management of bedding to maintain properties such as dryness, is also an important consideration. According to Rowbotham et al. [7], providing new bedding to stalls more than once weekly can reduce the bulk milk somatic cell score. This may have a positive benefit for the farm, because increased somatic cell count is related to milk loss [8].

The effect of bedding on dairy cows’ hygiene, udder health and milk quality differ between bedding material type. Manure solids used as bedding have high bacterial counts, leading to dirtier udders and higher coliform and streptococci counts than when sand or organic non-manure materials are used [9]. The diversity and abundance of the milk microbiota is affected by the environment where cows are housed (including their bedding [10]), and milked (with milk microbiota being similar to the microbiota on teat liners and teat dip cups [11]). High bacterial counts in bedding translate to high bacterial counts on cows’ teats, adversely affecting udder health and milk quality [5,12,13]. Sand used as bedding has several welfare advantages: as an inert substance, it does not support bacterial growth, and therefore it reduces the prevalence of mastitis [5]. It also has a lower surface temperature compared to wheat straw, rice husk and sawdust, which is beneficial in hot weather [14]. While it is important to consider microbial load in bedding materials, it is important to weigh this against the comfort and preferences of cows using these materials. Sand is not as comfortable as straw and hence cows lie down on sand for less time [15]. These effects on cows may be caused by differences in the physicochemical properties of the bedding materials [16,17].

Bedding materials that have traditionally been used in Chinese dairies include manure solids, sand, wheat straw, rubber mats, rice husks, and sawdust. Organic materials (e.g., wheat straw and dry sawdust) tend to improve the welfare of cows compared to inorganic materials (e.g., sand) [15,18]. However, for some dairy farms in China, the cost of recycled manure solids is prohibitive, and producers prefer to purchase other bedding materials. In Pennsylvania, USA, one survey found that bedding costs comprise 5% of the total cost of rearing replacement heifers [19]. In recent years, rising prices of raw materials have caused dairy farmers to consider alternative materials, including forest biomass (e.g., tree bark and vegetal fibers), conifer forest litter, seagrass (e.g., *posidonia oceanica*), and flax straw [17,20]. In China, peanut shells are a possible bedding material for dairy cows, supporting cow cleanliness to at least as high a degree as rice husk, which is another alternative bedding shown to be preferred by cows for lying on [21]. Despite this, the use of peanut shells and rice husk bedding in dairies has received little empirical scrutiny.

Scientific studies of the characteristics of bedding materials typically focus on the physical (e.g., dry matter content and water holding capacity) and, chemical properties (pH, total organic carbon, the C: N ratio, which may be important for compost-bedded pack) of the bedding, as well as microbial load (e.g., content of *Pseudomonas aeruginosa*, *Escherichia coli*, and *Streptococcus agalactiae*) [14,17,20]. Effect of bedding types on milk microbiota composition should also be explored. The present study aimed to evaluate the physicochemical properties of three bedding types (peanut shells, rice husks and a peanut–rice combination) and their composition of colostrum microbiota for use as bedding for dairy cows.

## 2. Materials and Methods

This study was conducted at the research dairy farm of Henan Agricultural University, in Zhengzhou, China, between January and April 2020. Seven primiparous and five multiparous (0 to 3 lactations), nonlactating and pregnant Holstein cows (mean 38 ± 11 d before parturition) between 2 to 6 years old, from the research dairy were used. Before the experiment, all cows were housed in one barn and bedded with dry manure. Cows were spilt into three bedding treatments with 4 cows per treatment. Each treatment comprised of two pens with the same bedding materials. Cows of each pen were divided by parity and calving date (two primiparous heifers in the last pen). The resting area in each pen was 5.9 m long and 5.15 m wide with a curb height of 15 cm. Cows were presented with one bedding material per pen. The three bedding treatments were with a sand base plus one of the following bedding materials to a depth of 10 cm: (1) peanut shells; (2) peanut–rice combination (a ratio of two parts of peanut shell to one part of rice husk by weight); (3) rice husks. The average time of cows kept in different bedding treatments was 32 d for peanut shells, 28 d for the peanut–rice combination, and 31 d for rice husks before calving. The effects of bedding types on cow behavior and welfare have already been reported and the management of the bedding has already been described [21]. In brief, the bedding was levelled daily, and feces removed while the cows were eating in their pen. An average 30 kg of fresh bedding material was added in each pen once weekly. Mean daily temperature inside the barn ranged from −1.2 to 18.1 °C measured using two portable weather stations recording at 10 min intervals.

### 2.1. Physical, Chemical and Biological Property Analyses

Surface samples of bedding materials were collected on days 0, 5, 13 and 20 for physical, chemical and biological analysis after bedding use. Analyses of the bedding materials were carried out in the Animal Husbandry Laboratory of Henan Agricultural University. The physical (e.g., dry matter and water holding capacity), chemical (e.g., pH level) and biological (e.g., *Streptococcus agalactia* content, *Pseudomonas aeruginosa* content and *Escherichia coli* content) properties of the bedding materials were analyzed.

To measure the dry matter (DM) content of the bedding, 200 g bedding samples per bedding treatment were collected in the sampling period. Each sample consisted of 5 subsamples (approximately 40 g per subsample) of the surface materials following the procedure described by Li et al. [21]. The samples were dried for 72 h at 65 °C using an electric heating constant temperature (blast) drying oven (DHG-9030A, Shanghai Jinghong Experimental Equipment Co., Ltd., Shanghai, China), and the percentage of dry matter content (%) was calculated using equation (1)

(1) $$\mathrm{DM}=\frac{M_{wet}}{M_{dry}}\times 100$$

where *M_wet_* is the mass of the new material (g), *M_dry_* is the dry mass of the material (g).

To measure water holding capacity (WHC), samples were divided into 10 g sub-samples per sample. They were first packed into a nylon bag, and then soaked in distilled water for 1 h. Any surface moisture was wiped from the nylon bag and it was then left on an iron grid for 30 min until water had ceased dripping from the bag. The water holding capacity was calculated from Equation (2). To measure pH levels of each bedding type, bedding material was mixed with water in a ratio of 1:10 based on weight for 30 min. A handheld pH meter (Testo 206 PH1, Testo International Trade (Shanghai) Co., Ltd., Shanghai, China) was then used to measure the pH level of the resulting solution.

(2) $$\mathrm{WHC}=\frac{M_{w}-M_{d}}{M_{wet}},$$

where *M_w_* is the mass of the saturated material and nylon bag (g) and *M_d_* is the dry mass of the material and nylon bag (g), *M_dry_* is the mass of the new material (g).

The bacterial content of bedding materials was determined by polymerase chain reaction (PCR). The genomic DNA of the bacteria in the bedding was extracted using a stool genomic DNA extraction kit (DP328, Tiangen Biochemical Technology (Beijing) Co., Ltd., Beijing, China). The abundance and purity of the genomic DNA obtained were detected using a NanoDrop One spectrophotometer (Thermo Fisher Scientific, Waltham, MASS, USA). Specific primers (Table 1) for genomic DNA were designed and synthesized by Sheng-gong Bioengineering (Shanghai, China) Co., Ltd. These primers were used to amplify bacterial DNA by PCR. Real-time PCR was performed using the LightCycler^®^96 (Roche, Basle, Switzerland) in 50 µL reaction volumes containing 25 µL of SYBR^®^ Premix Ex Taq™ II (2×; TaRaKa, Dalian, China), 1 µL of cDNA, 2 µL of forward and reverse primers, and 22 µL of RNase-free water. The PCR product was purified, recovered and cloned into the PMD18-T vector in 10 µL connection volumes containing 1 µL of PMD-19T, 5 µL of solution I, Vµl of DNA (V = 0.3 × 0.66 × the product length of gene/the concentration of the PCR product), up to 10 µL of RNase-free water, and the combination was then transformed into E. coli. Trans5a competent cells in accordance with the instructions of the manufacturer. The transformed product was added to a liquid medium containing antibiotics (Ampicillin Sodium) and incubated at 37 °C for 21 h. To check for a single colony in the culture medium, DNA sequencing at Sheng-gong Bioengineering (Shanghai, China) Co., Ltd., and DNAMAN software was used to compare the sequencing results with the target gene sequence on the NCBI website. A single colony of the bacteria was confirmed at similarity > 96%. The successfully transformed monoclonal positive bacteria had plasmid DNA extracted using endotoxin-free plasmid small extraction kit, and resulting samples were stored at −20 °C. Plasmid DNA was diluted in a 5-fold gradient for the preparation of calibration curves. The bacteria and plasmid DNA were detected by fluorescence quantitative PCR. Real-time PCR was performed using the LightCycler^®^96 (Roche, Basle, Switzerland) in 20 L reaction volumes containing 10 µL of SYBR^®^ Premix Ex Taq™ II (2×; TaRaKa, Dalian, China), 2 µL of cDNA, 2 µL of forward and reverse primers, and 6 µL of RNase-free water. The thermal cycling conditions were as follows: 5 min at 95 °C for one cycle, 35 PCR cycles (30 s at 95 °C, 30 s at 60 °C and 32s at 72 °C) and 10 min at 72 °C. This was followed by melt curves at 95 °C for 10 s and 60 °C for 5 s; cooling at 50 °C for 30 s. Reactions were stored at 4 °C. The cycle threshold (Ct) value of plasmid DNA was converted to copy number using the following formula: Copies·g^−1^ = DNA concentration (ng·µL^−1^) × 6.02 × 10^14^/(sum of base pairs of vector and PCR product)/650.

### 2.2. 16S rDNA Amplicon Sequencing of Colostrum Microbiota

Sampling the colostrum of cows that were housed on the three bedding types was undertaken in the milking parlor within 24 h of calving. For all cows, teats were wiped using a clean cloth towel and sanitized by iodine pre-dip. The first 3 streams of milk were not collected and the next 10 mL of milk per teat was poured into a sterile 50 mL tube. Microbial DNA was extracted from colostrum samples using HiPure Soil DNA Kits (Magen, Guangzhou, China) according to manufacturer’ protocols. The V3–V4 region of the 16S rDNA was amplified using primers 341F (CCTACGGGNGGCWGCAG) and 806R (GGACTACHVGGGTATCTAAT). The 16S rDNA target region of the ribosomal RNA gene was amplified by PCR (95 °C for 5 min, followed by 30 cycles at 95 °C for 1 min, 60 °C for 1 min, and 72 °C for 1 min and a final extension at 72 °C for 7 min) using primers 341F and 806R. Amplicons were extracted from 2% agarose gels and purified using the AxyPrep DNA Gel Extraction Kit (Axygen Biosciences, Union City, CA, USA) according to the manufacturer’s instructions and quantified using ABI StepOnePlus Real-Time PCR System (Life Technologies, Foster City, CA, USA). Purified amplicons were pooled in equimolar amounts and paired-end sequenced (PE250) on an Illumina platform according to standard protocols.

Raw reads were further filtered using FASTP (version 0.18.0, Chinese Academy of Sciences, Shenzhen, China). Paired end clean reads were merged as raw tags using FLSAH (version 1.2.11, Johns Hopkins University School of Medicine, MD, USA) with a minimum overlap of 10 bp and mismatch error rates of 2%. Noisy sequences of raw tags were filtered under specific filtering conditions to obtain the high-quality clean tags. The clean tags were clustered into operational taxonomic units (OTUs) of ≥97% similarity using UPARSE (version 9.2.64) pipeline. All chimeric tags were removed using the UCHIME algorithm and finally effective tags were obtained for further analysis. The tag sequence with the highest abundance was selected as a representative sequence within each cluster.

### 2.3. Statistical Analysis

Samples of bedding material were considered the experimental unit. Assumptions of normality and homoscedasticity were checked using the Shapiro–Wilk test and Levene’s test, respectively, in SPSS. Bacterial count data of bedding were base-10 log-transformed before analysis. There was no correlation between sampling time in GLM covariance using initial data as a covariant. Properties of bedding material were analyzed using a one-way ANOVA with SPSS (v.26, IBM, Armonk, NY, USA). The bedding treatment was included as an independent variable; all other variables as a dependent variable. When a significant effect was found, the Tukey post hoc test was used to determine differences between treatment means. For analysis of colostrum microorganisms, Chao1, ACE, Shannon, Simpson, Good’s coverage was calculated in QIIME (version 1.9.1) [22]. Alpha index among groups was calculated by Tukey’s HSD testing R Project Vegan package (version 2.5.3) [23]. Principal Coordinates Analysis (PCoA) based on the weighted unifrac distances was generated in R Project Vegan package (version 2.5.3) and plotted in R project ggplot2 package (version 2.2.1) [24]. The experimental unit was the colostrum sample of microbiota analysis. Figures were generated with GraphPad Prism 8 software (GraphPad Software, Inc., Sacramento, CA, USA). A statistical difference was assumed at *p* Value < 0.05.

## 3. Results

### 3.1. Physical and Chemical Property Analyses

Properties of bedding material are shown in Table 2. The dry matter content for peanut shells, the peanut–rice combination and rice husks were 73.3%, 78.6%, and 79.3%, respectively. Bedding dry matter was lower for peanut shells than for rice husks but did not differ from the peanut–rice combination. Water holding capacity and pH level did not differ between bedding treatments.

### 3.2. Biological Property Analyses

**Figure 1:**
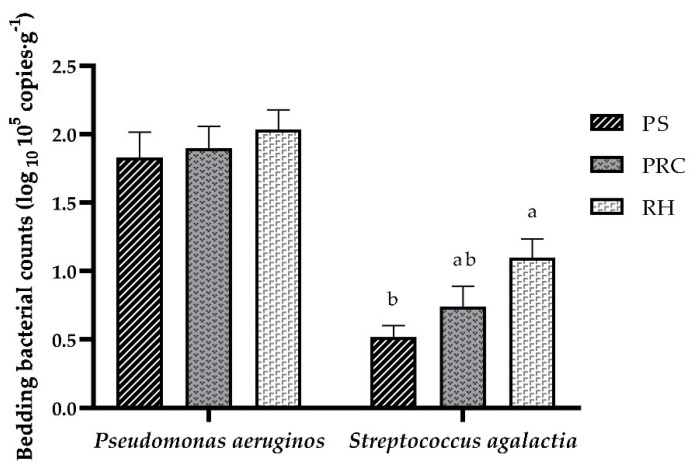
Counts of *Pseudomonas aeruginos* and *Streptococcus agalactia* in the bedding materials. PS = peanut shells, PRC = peanut–rice combination, RH = rice husks. Means ± SEM with different letters (a, b) differ (*p* < 0.05).

In this experiment, three bedding bacteria were tested. The phoA gene of Escherichia coli was not detected. For the other two bacteria, Pseudomonas aeruginosa and Streptococcus agalactia were found in all bedding samples (Figure 1). No differences between bedding materials were found for Pseudomonas aeruginosa. Streptococcus agalactia counts from peanut shell bedding was lower than from rice husks but did not differ from the peanut–rice combination.

### 3.3. Microbial Flora Analysis for Colostrum of Dairy Cows Housed on Three Bedding Materials

**Figure 2:**
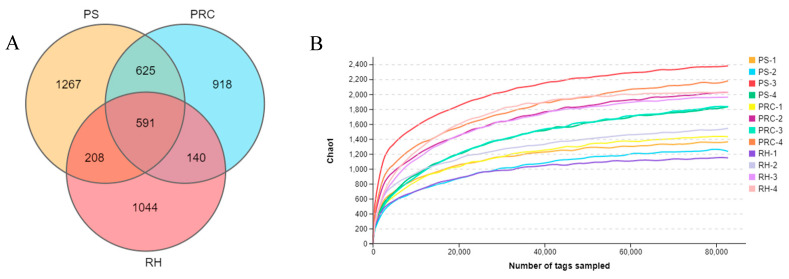
Venn diagram showing the number of unique and shared operational taxonomic units (OTUs) (**A**). High-throughput sequencing tag numbers and sequencing depth (**B**). PS = peanut shells, PRC = peanut–rice combination, RH = rice husks.

Twelve cases of bacterial 16S rDNA amplification products in colostrum were subjected to high-throughput sequencing. A total of 488,574, 496,487 and 478,391 original sequences were obtained for peanut shell, the peanut–rice combination and rice husk, respectively, and the effective sequences were 473,373, 483,123 and 452,088, respectively. The sequences were clustered into OTUs based on 97% identity. After filtering the chimera, a total of 2691 OTUs, 2274 OTUs and 1983 OTUs were obtained in the three treatments. The number of 591 OTUs shared by the three treatments accounted for 12.33%, and the number of OTUs unique to peanut hulls, peanut–rice combination and rice husk accounted for 26.43%, 19.15% and 21.78% of the total, respectively (Figure 2A). Tag numbers and sequencing depth are shown in Figure 2B.

#### 3.3.1. Alpha and Beta Diversities of Microbiota from Colostrum Samples

The Chao 1 richness, ACE richness, Shannon and Simpson diversity of colostrum samples did not differ between the three bedding materials (*p* > 0.05) (Table 3). For Shannon diversity the range was 7.50 to 7.69 with a mean of 7.57, and for Simpson diversity the range was 0.97 to 0.98 with a mean of 0.98.

**Figure 3:**
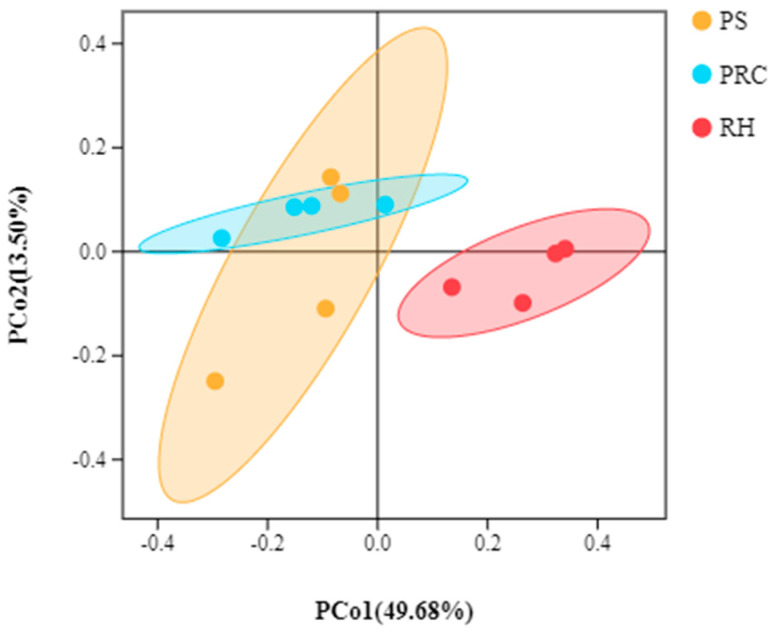
Principal coordinates analysis (PCoA) plot of colostrum samples. PS = peanut shells, PRC = peanut–rice combination, RH = rice husks. Overlap within two habitats indicated that exposure to different bedding types had similar bacterial community compositions of colostrum samples, if not the bacterial community was different.

PCoA is a dimensionality reduction analysis based on a distance matrix, which evaluates the degree of explanation of the overall difference in bacterial colony structure by each coordinate axis as a percentage. In the analysis results, the more similar the samples are, the closer the distance is reflected in the PCoA. PCoA analysis of the colostrum of cows housed on different bedding materials indicated differences in bacterial community composition, although there were similar levels of richness and diversity (Figure 3). A large overlap was observed between the colostrum from cows on peanut shells and those on the peanut–rice combination, but separate clusters were observed between the colostrum from cows on rice husks samples and peanut shells or the peanut–rice combination.

#### 3.3.2. Composition of Colostrum Microbiota

**Figure 4:**
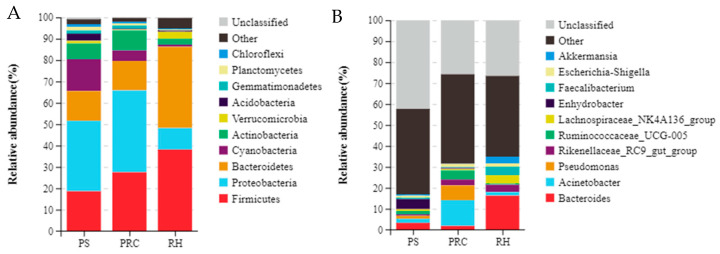
Bacterial composition in colostrum at the phylum (**A**) and genus (**B**) level for dairy cows from different bedding materials. PS = peanut shells, PRC = peanut–rice combination, RH = rice husks.

Across the three groups, *Proteobacteria* (33.0%), *Firmicutes* (18.6%), *Cyanobacteria* (14.9%), *Bacteroidetes* (13.9%) and *Actinobacteria* (7.3%) were the five most abundant bacterial phyla in colostrum from cows bedded on peanut shells. *Proteobacteria* (38.3%), *Firmicutes* (27.5%), *Bacteroidetes* (13.8%), *Actinobacteria* (9.3%) and *Cyanobacteria* (4.9%) were the five most abundant bacterial phyla in colostrum from cows bedded on the peanut–rice combination. *Firmicutes* (38.1%), *Bacteroidetes* (38.0%), *Proteobacteria* (10.1%), *Verrucomicrobia* (3.4%) and *Actinobacteria* (2.7%) were the five most abundant bacterial phyla in colostrum from cows bedded on the rice husks (Figure 4A). Colostrum from cows on the peanut shells had higher (*p* < 0.05) *Proteobacteria*, *Actinobacteria* and lower (*p* < 0.05) *Bacteroidetes*, *Verrucomicrobia* and *Epsilonbacteraeota* compared with rice husks, but did not differ from the peanut–rice combination.

At the genus level, the predominant bacterial genera in colostrum were *Enhydrobacter* (4.9%), *Bacteroides* (3.3%) and *Acinetobacter* (2.0%) for peanut shell bedding, *Acinetobacter* (12.3%), *Pseudomonas* (7.1%), *Ruminococcaceae_UCG-005* (4.3%) and *Rikenellaceae_RC9_gut_group* (2.8%) for the peanut–rice combination bedding, and *Bacteroides* (16.3%), *Lachnospiraceae_NK4A136_group* (4.1%), *Faecalibacterium* (3.9%) and *Rikenellaceae_RC9_gut_group* (3.4%) for rice husk bedding (Figure 4B). Colostrum of cows bedded on peanut shell had a higher abundance of *Enhydrobacter* and *Pantoea* of colostrum microbiota (*p* < 0.05). For colostrum of cows on the peanut–rice combination, proportions of *Pseudomonas* and *Corynebacterium_1* were higher compared with those in peanut shell or rice husk. Relative abundances of *Bacteroides*, *Akkermansia*, *Alistipes*, *Ruminococcaceae_UCG-014*, *Coriobacteriaceae_UCG-002* and *Intestinimonas* were significantly higher (*p* < 0.05) for cows on rice husk.

## 4. Discussion

The present study was to evaluate the physicochemical properties of three organic materials as bedding for dairy cows, and to understand the effect on colostrum microbiota of these cows. Our results indicate that peanut shells may be promising bedding material for dairy cows. In this study, a difference in dry matter between bedding was observed. Higher dry matter content was found in rice husks bedding compared to peanut shells. The dry matter of bedding materials is an important factor affecting bacterial counts and cow comfort. Cows usually prefer to lie down on a dry surface, and the wet bedding material impairs their welfare by affecting their health and lying time. Higher bacterial counts were detected in manure solids with lower dry matter content reported by Patel et al. [9], which is similar to that reported by Wolfe et al. [25]. Additionally, the wet bedding can dirty the udder of cows, which may increase mastitis risk [3,9]. On the other hand, Reich et al. [26], reported that the lying time of cows is 10.4h/d on wet bedding (34.7% DM) compared to 11.5 h/d on dry bedding (89.8% DM). According to Reich et al. [26], the lying time of cows remains stable when the dry matter content of bedding is above 62.2%. In this experiment, the dry matter of all three bedding types were above 73.3%. Rice husks had the highest dry matter content at 79.3%, followed by the peanut–rice combination (78.6%). The bedding materials in our study can be used for dairy cows, and rice husk bedding was superior in dry matter content. 

In the current study, the pH value was not affected by bedding treatment. *Pseudomonas aeruginosa* can survive at 5.6–9.0 pH but the maximum growth observed is between 6.6–7.6 The highest biofilm formation rate of *Streptococcus agalactiae* is at pH 6.5 [27]. Our mean pH for rice husks of 9.2 was in line with the published report by Patel et al. [9,14], who found the pH was 8.7 for rice husks. A low pH level of bedding materials promoted bacterial growth. After use, the pH of peanut shells and the peanut–rice combination bedding in the study increased to above 9.0 but pH for rice husks was high throughout. This change may be attributed to ammonia from urine and differences in properties of the beddings [14]. Quality bedding material needs to absorb moisture, maintain dryness, and allow animals to display natural behaviors [28]. Water holding capacity is an important property of bedding material as it shows the moisture content that the material absorbs and stores [28]. Additionally, Ahn et al. [29], reported that the water holding capacity of materials increases with increasing moisture content. Similar water holding capacity of three bedding materials were found in this study. Our results agree with those of Ferraz et al. [17], who reported that the water holding capacity was 1.62 for barley husks and 1.65 for spelt husks. However, Ferraz et al. [17], found that *Posidonia oceanica* (7.32) in wood shavings (4.89) and barley straw (4.13) had higher values compared with ours. This may be attributed to the moisture, texture, and structure of the materials [30]. 

Compared with non-organic material, organic bedding provides a more comfortable surface, improvement in cow welfare, and is favored by cows. However, the high load of pathogenic bacteria in organic material is a concern for farmers. *Escherichia coli*, *Streptococcus agalactiae*, *Klebsiella spp.*, *Pseudomonas aeruginosa* and *Staphylococcus aureus* are common microorganisms that endanger the health of cow udders and cause mastitis [31,32,33]. Major reductions are observed in performance (cumulative milk, fat and protein yield) and gross profit for cows with mastitis [34,35]. Cows with mastitis also have a decreased percentage of pregnancies for first artificial inseminations and increased pregnancy loss compared with healthy cows [36]. Bedding materials to which cows’ teats are exposed have significant effects on the prevalence of mastitis [37,38]. Robles et al. [5], found that, bedding material of manure solids presented higher gram-negative bacterial counts compared with sand, straw and wood shavings, indicating that there are differences in bacterial counts between different materials. Consequently, reducing bacterial counts on the surface of bedding is important to reduce the risk factor of mastitis of cows. In the current study, the presence of *Pseudomonas aeruginosa* and *Streptococcus agalactia* was noted in all bedding materials. No differences were observed on *Pseudomonas aeruginosa* counts between bedding types, although they were lower in peanut shells. However, peanut shells had lower *Streptococcus agalactia* counts compared with rice husks. Our results agree with those of Fávero et al. [39], that peanut shells have lower bacterial counts. This illustrated that bacterial counts were better controlled in peanut shells than in rice husks, and had a minimal risk of udder infection for peanut shell bedding.

The proportions of OTUs in the milk of cows kept on different bedding is likely to differ [40]. Wu [10] and Nguyen [41] et al., reported that milk microbiotas are associated with both the bedding and airborne dust microbiota. Bacteria types and counts on the teats of cows are affected by bedding types, and manure solids with high bacterial counts cause more *Escherichia coli* in the milk tank [9,40,42]. These papers indicate that differences in milk microbiota may be attributed to the environment in which the cows are housed, especially the teat dip cup, airborne dust and bedding types directly exposed to teats. In the current study, no significant differences were found in Alpha diversities of the colostrum microbiota of cows on different bedding types, which is similar to the report by Metzger et al. [40]. With regard to PCoA of milk microbiota, samples from cows housed on peanut shell bedding had a large overlap with the peanut–rice combination, but differed from the cows bedded on rice husks. Metzger et al. [40], reported that there were differences in milk microbiota composition for cows kept on manure solids, recycled sand, sawdust and new sand. It has been demonstrated that the milk microbiota is a dynamic community with the shift of environment [11,40,41,43]. Nguyen et al. [44], found that differences in milk microbiota derived from two farms at the family level. In our study, bedding treatments did affect the relative abundance of the prevalent bacteria, e.g., *Firmicutes*, *Bacteroidetes*, *Proteobacteria* and *Actinobacteria*. The proportion of dominant bacteria was similar to the report that *Proteobacteria* and *Firmicutes* were two prevalent phyla by Nguyen et al. [44]. Ganda et al. [45] reported that the milk microbiota from healthy cows were also dominated by *Proteobacteria* and *Firmicutes*. In the current study, different bedding materials (pens with different bedding types can be considered as a distinctive environments) provided a potential difference in colostrum microbiota at the phylum level. Similarly, differences of dominant and non-dominant bacteria in colostrum samples were observed at the genus level. Significant enrichment for *Enhydrobacter* and *Pantoea* in the colostrum of cows housed on peanut shells were found. *Enhydrobacter* and *Pantoea* belong to gram-negative bacteria. According to Aldrete-Tapia [46], and Metzger et al. [47], *Enhydrobacter* is considered to be the subdominant genus with a relative abundance of 0–21.52%, which varies with season. *Pantoea* has been confirmed to exist in the barn and has biological control activity against fungal pathogens [48,49].

Colostrum samples from cows using the peanut–rice combination had greater relative abundance of *Pseudomonas* and *Corynebacterium*. *Corynebacterium* is an exogenous pathogen, belonging to gram-positive bacteria, which mainly causes subacute inflammation through udder trauma [50,51]. *Psychrophilic* bacteria of the genus *Pseudomonas* have been found to cause milk spoilage. However, the *Pseudomonas* bacterium is not unique in healthy milk; it is also the only bacteria that exhibits a random distribution in milk microorganisms and is usually inhibited by the probiotic network in healthy milk cows [52,53]. However, *Pseudomonas* which was significantly enriched in colostrum of cows housed on the peanut–rice combination, as a potential pathogen, is still worth noting as a risk factor for udder health. An increased prevalence of *Bacteroides*, *Akkermansia*, *Alistipes*, *Ruminococcaceae_UCG-014*, *Coriobacteriaceae_UCG-002* and *Intestinimona* in colostrum of cows on rice husks was found. In this experiment, our results that the highest abundance for *Bacteroides* was in the colostrum of cows on rice husks is similar to the report by Oikonomou et al. [54]. *Akkermansia*, belonging to the phylum *Verrucomicrobial*, is considered to be a beneficial microorganism and is negatively related to certain metabolic disorders [55]. *Alistipe* of the phylum *Bacteroides* is not present in abundance in milk with mastitis compared with healthy milk [52]. Derakhshan [50], and Doyle et al. [56], reported that *Ruminococcaceae*, as the dominant flora in colostrum, is easily affected by the environment. No studies have been reported of *Coriobacteriaceae_UCG-002* and *Intestinimonas*. A significant enrichment for *Corynebacterium* and *Pseudomonas* in the colostrum of cows using the peanut–rice combination may be an increased risk factor for mastitis compared with other beddings. Adverse risks factors for the colostrum microbiota of cows using peanut shells and rice husks have not been observed.

## 5. Conclusions

Based on the results of these experiments, differences in the bacterial community composition of colostrum were affected by bedding types. The physicochemical properties exhibited small differences among the bedding materials, except for dry matter content. However, peanut shell bedding reduced bacterial growth and had no effect on colonization of colostrum by mastitis pathogens which illustrates that it appears to be a potential bedding material for dairy cows. Further research is needed to determine more properties of peanut shells and rice husks, including particle size, bulk density and cost.

## Figures and Tables

**Table 1:** The primer sequence of the genes and the length of the product.

Bacteria	Gene Name	Primer Sequence (5′-3‘)	Annealing Temperature°C	Length bp
*Streptococcus agalactia*	ef-tu	ACCACGAAGAAGAACACC	52	515
		GACCAATGCCACAAACTC		
*Pseudomonas aeruginosa*	MS6	AGACACGGTCCAGACTCCTAC	58	277
		CCAACTTGCTGAACCACCTAC		
*Escherichia coli*	phoA	GGTAACGTTTCTACCGCAGAGTTG	58	468
		CAGGGTTGGTACACTGTCATTACG		

**Table 2:** Physicochemical properties of the bedding materials.

	Bedding Treatment ^1^					
Item	PS	PRC	RH	SEM	Test Statistic	*p*-Value
DM ^2^, %	73.3 ^b^	78.6 ^ab^	79.3 ^a^	1.05	F_(2,47)_ = 3.751	0.032
pH	8.9	9.0	9.2	0.08	F_(2,59)_ = 0.819	0.446
WHC ^3^	1.6	1.5	1.5	0.02	F_(2,46)_ = 0.690	0.507

^1^ PS = peanut shells, PRC = peanut–rice combination, RH = rice husks. ^2^ DM = dry matter. ^3^ WHC = water holding capacity. Different superscript letters within a row indicate *p* < 0.05.

**Table 3:** Chao 1 richness, ACE richness, Shannon and Simpson diversity of colostrum samples.

**Item**	**PS**	**PRC**	**RH**	***p*-Value**
Chao1	1778.54	1944.56	1691.60	0.69
ACE	1828.33	2018.78	1767.85	0.69
Shannon	7.52	7.69	7.50	0.84
Simpson	0.97	0.98	0.99	0.58

PS = peanut shells, PRC = peanut–rice combination, RH = rice husks. Chao1/ACE index mainly cares about species richness information of samples. Simpson/Shannon mainly comprehensively reflects the richness and evenness of species.

## Data Availability

The data presented in this study are available on request from the corresponding author.
